# BIGSdb: Scalable analysis of bacterial genome variation at the population level

**DOI:** 10.1186/1471-2105-11-595

**Published:** 2010-12-10

**Authors:** Keith A Jolley, Martin CJ Maiden

**Affiliations:** 1 Department of Zoology, University of Oxford, South Parks Road, Oxford, OX1 3PS, UK

## Abstract

**Background:**

The opportunities for bacterial population genomics that are being realised by the application of parallel nucleotide sequencing require novel bioinformatics platforms. These must be capable of the storage, retrieval, and analysis of linked phenotypic and genotypic information in an accessible, scalable and computationally efficient manner.

**Results:**

The Bacterial Isolate Genome Sequence Database (BIGSDB) is a scalable, open source, web-accessible database system that meets these needs, enabling phenotype and sequence data, which can range from a single sequence read to whole genome data, to be efficiently linked for a limitless number of bacterial specimens. The system builds on the widely used mlstdbNet software, developed for the storage and distribution of multilocus sequence typing (MLST) data, and incorporates the capacity to define and identify any number of loci and genetic variants at those loci within the stored nucleotide sequences. These loci can be further organised into 'schemes' for isolate characterisation or for evolutionary or functional analyses. Isolates and loci can be indexed by multiple names and any number of alternative schemes can be accommodated, enabling cross-referencing of different studies and approaches. LIMS functionality of the software enables linkage to and organisation of laboratory samples. The data are easily linked to external databases and fine-grained authentication of access permits multiple users to participate in community annotation by setting up or contributing to different schemes within the database. Some of the applications of BIGSDB are illustrated with the genera *Neisseria *and *Streptococcus*.

The BIGSDB source code and documentation are available at http://pubmlst.org/software/database/bigsdb/.

**Conclusions:**

Genomic data can be used to characterise bacterial isolates in many different ways but it can also be efficiently exploited for evolutionary or functional studies. BIGSDB represents a freely available resource that will assist the broader community in the elucidation of the structure and function of bacteria by means of a population genomics approach.

## Background

Parallel sequencing technology, sometimes referred to as 'next-generation' sequencing, makes possible the rapid determination of large numbers of complete bacterial genome sequences at a low cost. This is leading increasingly to its use in population studies including epidemiological investigations, a trend that will accelerate with the continual introduction of technical advances such as single molecule sequencing [1]. The possibility of comparing any number of gene targets among multiple, disparate, isolates, allows the assembled data resource to be used to address a wide range of research questions concerning bacterial evolution, ecology and pathogenicity. Harnessing this resource will enable a diversity of information to be efficiently exploited in functional studies.

Investigations of bacterial population biology and epidemiology have utilised whole genome data but, until now, its application has been limited to largely clonal organisms or closely related isolates [2-8]. In order to facilitate wider bacterial population genomic research, there is a need to link whole genome data to the population sample data, including detailed provenance, clinical information and phenotype as appropriate, allowing integrated studies irrespective of the diversity of the isolates. One method that has been widely used to achieve this for both population studies and especially epidemiological investigation is multilocus sequence typing (MLST) [9,10]. MLST indexes the sequences of representative housekeeping gene fragments, approximately 500 bp in length usually from seven loci, with each unique allele assigned an arbitrary integer identifier. Unique combinations of the alleles at each locus, allelic profiles, are identified by a sequence type (ST) number with definitions stored in authoritative online databases overseen by a curator for each species or group of species. Housekeeping gene profiles may be combined with sequence data from more rapidly-evolving genes, such as those encoding surface antigens, where higher resolution is required to address questions concerning antigenic variation or antibiotic resistance [11,12]. The current generation of sequence typing databases [13-15] have been highly successful at linking isolate provenance to sequence data, contributing significantly to the widespread adoption of MLST and antigen sequence typing. Indexing population genomic data provided by parallel sequencing technologies, however, provides challenges requiring the development of new methodologies and informatics solutions.

We have extended the proven concept of MLST to genome scale data, where particular combinations of loci can be analysed depending on the question being addressed. As with conventional MLST, each unique sequence at each locus can be assigned an allele number, allowing the range of analyses developed for MLST, or for other techniques utilizing numerical profiles such as multiple locus VNTR analysis (MLVA) [16], to be directly applied to whole genomes, while still providing full access to the underlying sequence data when required. This approach provides the isolate- or specimen-centric view required for epidemiology, ecology and population analysis, with the isolate provenance and phenotype linked to any quantity of sequence data, from individual dye-terminator sequencing reads, through partial genomes consisting of multiple unassembled contigs to complete genome sequences. It also enables routine typing applications for epidemiological studies to use the same methodology as required for population analysis, which has been one of the factors in the success and widespread adoption of MLST. While analogous to MLST, the application of the concept goes far beyond typing, allowing detailed investigation of population diversity in bacterial systems. Here we describe a software platform for population genomics that has been designed and developed to exploit this concept.

## Implementation

BIGSDB is written in Perl for installation on UNIX/Linux systems. It utilizes the PostgreSQL database and Apache web server software running under mod_perl [17] to avoid the performance penalty of Perl interpreter start-up times. Sequence handling routines are provided by the BIOPERL library [18] and EMBOSS suite of programs [19]. Client-side Javascript makes use of the JQUERY library [20]. Built-in authentication is based on Perl/Javascript MD5 secure user authentication [21], with Javascript MD5 code written by Paul Johnston [22]. Sequence homology matching uses BLAST[23] with a default word size of 15 and identity of 70% for DNA sequences (values are configurable by the user).

### Configuration

Global configuration settings are stored in a text file. Settings provide the locations of helper applications like BLAST and EMBOSS and the names of the preference and authentication databases. Logging utilizes the Log::Log4perl module, enabling fine-grained control of error and status logging by modifying a configuration file, setting the overall log level for discrete components of the system.

Individual databases are configured with a XML file that describes any isolate provenance fields including default display properties (that can be over-ridden by user preferences), sample table for use as a LIMS database, connection information, web paths, authentication type in use and enabled plug-ins.

### Sequence definition databases

As well as storing isolate information, BIGSDB is able to host separate sequence definition databases so that new allele sequences can be defined for any locus and made available on the Internet. Users are able to paste in and query sequences against all known alleles from a particular locus or against all loci. The nearest matches are displayed along with nucleotide differences and the start and end positions within the sequence are identified, allowing exact polymorphisms to be identified and checked rapidly and efficiently without the user having to trim their sequence to match the defined allele.

Sequence definition databases can also define schemes so can, for instance, make MLST profile data available.

### User customization

Individual users are able to customize the query interface. The fields and loci that are returned within main results tables following a query can be selected so that only the results of interest are shown without leading to the table width going beyond the confines of the page. Drop-down list boxes that filter search results based on particular provenance criteria or publications can be added to the standard query interface. All settings are stored in a separate preference database linked to a unique identifier stored as a HTTP cookie so that these are remembered between sessions.

### Data export

Isolate data, along with all defined allele identifiers, can be exported for a subset of the data returned from a query or for the whole database. Isolate data are exported in tab-delimited text format, suitable for importing into spreadsheets or easy parsing by automated scripts. Concatenated sequences can be exported for isolates in FASTA or extended-FASTA format suitable for use in third-party phylogenetic packages.

### Plug-in architecture

The software employs a plug-in architecture, allowing additional features and analysis packages to be added by third-parties without modification of the core code. Various attributes are defined for each plug-in which specify whether it works with isolate or sequence definition databases, where in the interface it should be available, e.g. main index page and/or following a query to utilise a returned dataset, and a feature category, e.g. export, breakdown, or analysis, allowing tools to be grouped by function.

Most software functionality that can be considered optional to the core requirements of the database form part of plug-ins, easing maintenance of the main code and allowing installations to be tailored to individual requirements. Since plug-ins are self-contained units, they can be distributed under different licenses to the main software package.

### Authentication and access control

There are three types of user in BIGSDB: i) 'users' can view data but never modify it; ii) 'curators' can add and modify data with specific permissions enabling particular roles to be defined and controlled; and iii) 'admins' have full control over the database structure, data and curator permissions.

The software can be configured with either built-in authentication or controlled by apache. Built-in authentication uses client-side Javascript to hash passwords together with session identifiers so that passwords are not transmitted in clear text over the network. Controlling authentication within the program also allows users to change their own passwords from the web interface. Using apache authentication allows any supported external authentication scheme to be employed.

Isolate databases can be configured to be public, where either everybody on a public website or all authenticated users can view all records. Alternatively, controls can be configured allowing read and write access of individual isolate records to specific users or user groups. By default, new records are viewable by everybody and writable by the curator who adds them, but access can be controlled easily within the curator interface in either a single isolate manner or batch mode. Curation access to individual loci in sequence definition databases can be set so that curators are allowed to define allele sequences for certain loci only. This allows a single definitions database to be serviced by a community of curators, expert in particular areas of the organism's biology.

## Results

### Design philosophy

**Figure 1:**
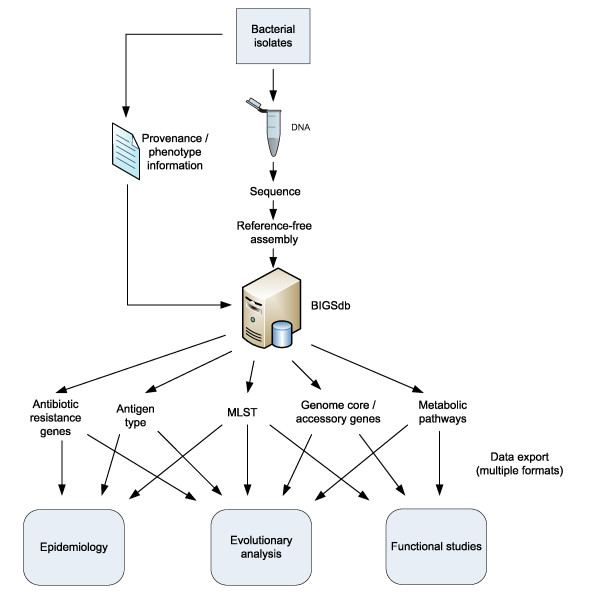
**BIGSdb links bacterial isolate provenance, phenotypic and genotypic data**. Sequences from multiple sources such as single dye-terminator reaction reads, contigs generated from parallel sequencing technologies or complete assembled genomes can be associated with an isolate record. Following locus tagging, sequences can be readily extracted and exported in formats suitable for various analyses.

**Figure 2:**
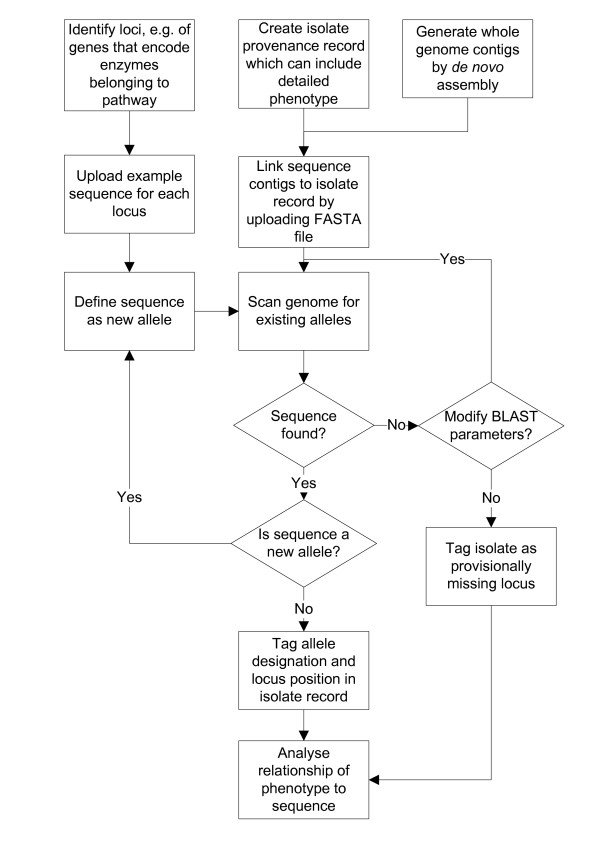
**Iterative process of analysing loci**. Since detailed phenotypic information can be included with the isolate record, the correlation between enzyme sequence diversity and phenotype can be examined using the integrated analysis tools.

The Bacterial Isolate Genome Sequence Database (BIGSDB) is an informatics system that can hold provenance and phenotype information on an unlimited number of isolates, along with nucleotide sequence data (Figure 1). These sequence data can be of any size scale ranging from individual dye-terminator sequencing reads through partial assemblies generated from parallel sequencing technologies to complete assembled genomes. These are stored within a 'sequence bin' linked to the isolate records. A reference sequence for each locus can be defined, or alternatively they can be linked to external databases that hold allele sequence definitions. This enables the positions of loci within individual sequences stored in the bin to be determined automatically using any algorithm, at present BLAST[23], and the sequences extracted along with flanking regions if required. If an external sequence definition database, containing allele sequences and their identifiers, has been defined it can automatically be queried to determine the allele number for each locus (Figure 2). BIGSDB facilitates the construction of these definition databases. Provided the locus is fully encompassed within a single contig, the length of individual sequences is unimportant for extracting allele data, avoiding many of the problems associated with assembly of short-read data.

Genetic loci can be grouped into schemes with membership defined by any criteria and with each unique combination of alleles associated with a primary key (a field that uniquely defines this combination) and any number of other fields. One example of this is the standard seven locus MLST scheme where each allelic profile is defined by a ST number, the primary key in this case. Since the ST can also define membership of a clonal complex, an epidemiologically related grouping of STs, this can be included as an additional field in the scheme. The flexibility of BIGSDB enables loci to belong to any number of schemes, allowing multiple strain nomenclatures to be cross-referenced or for schemes based on specific aspects of the biology of the organism to be employed, such as particular biochemical pathways, surface components coded by antigen genes, antibiotic resistance or members of macromolecular complexes.

### Allele assignment and locus tagging

As well as automatic allele assignment using BLAST, allele numbers can be assigned manually, enabling existing isolate datasets to be imported where these designations are already determined. Competing allele designations, identified by different users or with different analysis methods, can be handled with the first determined designation used for analysis, but the presence of conflicting data highlighted within the user interface. Designations can be promoted or demoted from the active or pending state by the curator; with a fine-grained permission system allowing specific curator roles (see access control in Methods). A full history of changes made to an isolate record is logged, so that it is possible to track which curator made a change and when.

Loci are usually defined by nucleotide sequences, but BIGSDB will also handle loci that are defined by the translated sequence peptide, commonly used, for example, to define variable regions of antigens important in typing or vaccine development. Irrespective of the locus type, the sequence definition database can be queried using either nucleotide or peptide sequences, with the query type recognized automatically and the appropriate BLAST algorithm called. Alleles can be named using either simple integers, or by a text string, with the format naming constrained by a regular expression defined in the locus table if required. Coding sequence definitions from existing published annotations can be retrieved automatically by entering a Genbank or EMBL accession number, assisting the process of setting up new loci for analysis.

### Isolate and locus aliases

A common issue with population datasets is that of alternative nomenclature of isolates, with many samples having multiple names having been stored in different laboratories or collections. BIGSDB allows isolates to have any number of names by storing aliases in a linked table. These aliases are treated in the same manner as the primary name, and will be found by searches against the 'isolate' field. In a similar manner, loci can also have aliases, all of which are accessible within the interface. Loci that are members of schemes defined within external databases are not constrained to the names used in those databases, ensuring that data organisation within BIGSDB is not impacted by external naming constraints.

### Data export and analysis

Sequence and provenance data can be exported from the database in multiple formats. Where genome data are represented by single or a small number of large contigs, export in EMBL format with the locus information included provides a method of consistent feature annotation, allowing newly defined loci to be applied rapidly to any number of existing genomes. Other formats include extended multi-FASTA where data for each locus are grouped in aligned blocks, facilitating whole genome phylogenetic analysis with applications such as ClonalFrame [24].

Datasets can be further analysed by provenance or allele content using various breakdown tools that determine value frequencies or that breakdown one field against another, allowing analyses such as clonal complex against serogroup.

### Laboratory Information Management System (LIMS)

BIGSDB can form the basis of a laboratory information management system (LIMS). An optional samples table can be defined containing any choice of fields which can, for example, describe sample type and freezer location. Each isolate record can then be associated with any number of samples which are displayed within the detailed isolate information pages, where the records can be updated by users with appropriate privileges.

### Demonstration 1: PubMLST and reference gene-based analysis of partially assembled genome data

We have installed BIGSDB on PubMLST.org and converted the *Neisseria *MLST databases [25] to use the system in place of the previously used MLSTDBNET[13] software, the functionality of which is generically incorporated. The MLST databases for *Neisseria *are the largest of all such databases, containing provenance and genetic data for over 17,000 isolates and over 8,000 STs, providing a valid test of scalability and performance for population level data. The isolate database has been linked to existing antigen typing and antibiotic resistance gene databases [26], enabling automated allele assignment and sequence tagging of typing antigens and genes encoding candidate vaccine proteins. We have further populated this database with publicly-available *Neisseria *species genome data and performed automated tagging and assignment of alleles for loci with existing definition databases. Some of these are represented by single contigs of approximately 2.2 Mbp, but there are also samples with unfinished genomes consisting of multiple smaller contigs. Finally, we have deposited contigs for isolate OX9932088, a ST-41/44 isolate collected during the UK meningococcal carriage study [27], generated from Illumina Solexa reads, to demonstrate the ease of analysis of such data using the gene reference approach [Neisseria PubMLST:14923]. Velvet assembly [28] of the Solexa runs yielded 295 contigs of >100 bp length, which were uploaded to the PubMLST BIGSDB database. Automated sequence tagging determined the full strain designation, B: P1.21,16: F1-5: ST-1415 (cc41/44), incorporating MLST and the PorA and FetA antigen types. Additionally, the peptide sequence for Factor H binding protein, a principal component of two meningococcal recombinant protein vaccines undergoing clinical trials, was identified as variant 19 [29]. Allele sequences for *penA *[30] and *rpoB *(definitions incorporated into PubMLST) were identified as *penA*-1 and *rpoB*-18 indicating the isolate has intermediate susceptibility to penicillin and high susceptibility to rifampicin respectively. Isolate records for which genome data are available can be readily extracted from the database by selecting 'whole genome' in the project filter of the query interface.

### Demonstration 2: Relationships within the genus *Streptococcus*

Since BIGSDB can define multiple schemes for a dataset, it can be used to cross-reference typing methods. A database containing 43 published streptococci genomes was constructed [31] and had loci defined for all streptococcal MLST schemes (*S. agalactiae *[32], *S. oralis *[33], *S. pneumoniae *[34], *S. pyogenes *[35], *S. suis *[36], *S. uberis *[37] and *S. zooepidemicus *[38]). Sequences and ST definitions from these schemes were imported into a unified definitions database, and the genomes tagged with all loci found. In addition, unique alleles from the streptococcal MLSA database [39], whose loci were chosen to be present across the viridans streptococci, were imported and assigned allele numbers. Genome isolates were then tagged against these loci as well.

**Figure 3:**
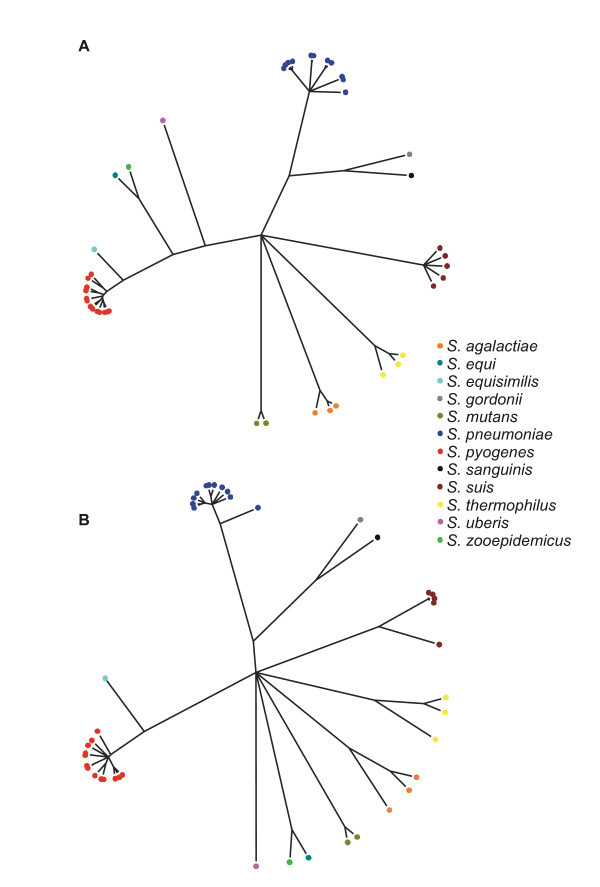
**The Genome Comparator plugin can identify loci shared among genomes**. ClonalFrame trees were generated from 43 Streptococcal genome sequences using A) seven MLSA gene fragment loci and B) 77 complete genes found to be present throughout the genus identified by BIGSDB. Aligned sequences were exported from the database and 50% consensus trees generated from six independent runs with 50 k iterations, 50 k burn-in iterations, and a thinning interval of 100.

Further loci were defined based on a sample of the coding sequences extracted from the annotated *S. equi *genome [40]. The BIGSDB genome comparator tool was used to identify loci found in all 43 genomes using BLAST with a 70% identity cut-off and a word size of 8. Because the search used nucleotide sequences it would be expected to only find the more conserved classes of protein-coding genes [41]. Seventy-seven coding loci, consisting largely of genes whose products are involved in translation were identified. Sequences for the MLSA scheme and the 77 trans-genus loci were then extracted as two separate datasets from the database as aligned sequences in multi-FASTA format using the BIGSDB export functionality. ClonalFrame trees were generated from these sequence data (Figure 3). The trees from the MLSA loci and from the 77 loci identified without *a priori *knowledge produced highly similar species clustering. The only major differences between the two trees was that the branch points for the *S. equi*/*S. zooepidemicus *and the *S. uberis *branches were positioned nearer to the *S. pyogenes *cluster in the MLSA tree.

## Discussion

### Flexible storage of population-scale bacterial genome data

**Figure 4:**
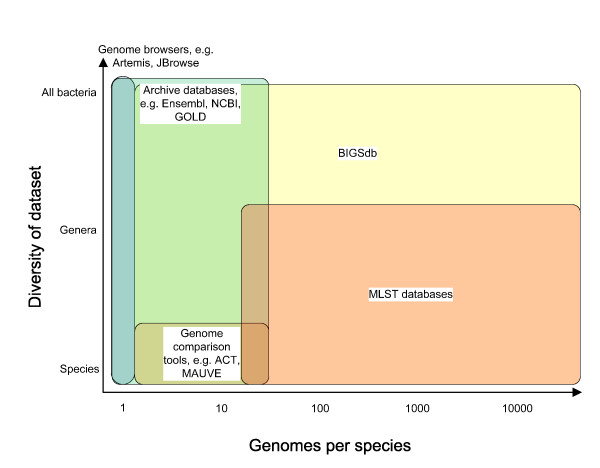
**The BIGSdb database platform is highly scalable**. The system can be used to analyse a few isolates up to many thousands, each with full genome data attached. This compares favourably with existing database and analysis resources.

There is a dichotomy in the approach to data handling and analysis between the researchers that have been involved in generating complete annotated genome sequences and those engaged in large scale bacterial population studies. To date, the former have worked with relatively few isolates as exemplars of their species, while the latter have collected and analysed datasets that may include hundreds or thousands of isolates with less complete genome sampling. Many of the genomes hosted in online databases [42-44] have detailed and comprehensive annotation, but what these databases have in common is that for any particular species they contain very few genomes (Figure 4) and the analysis is geared specifically to the attributes of the sequence itself rather than of the isolate from which it was derived. For example, data cannot be analysed based on host clinical outcome, geographical location or any number of attributes that are important in evolutionary or epidemiological studies.

Conversely, population biologists and bacterial epidemiologists collect and analyse many more isolates, but until now, have sampled the genome by sequencing relatively few genes. Methods of sampling a genome with manageable units of data that are epidemiologically meaningful will continue to be essential for characterisation of isolates and disease management and with the decreasing costs of parallel sequencing it is likely, in the near future, to be more efficient and cost-effective to obtain MLST and antigen profiles by sequencing the whole genome rather than by a gene-by-gene approach using dideoxy chain termination methods. MLST and antigen sequencing have proved sufficient for routine typing, and they have been used successfully for association studies that link host, disease or sample site to genetic type [45-49]. The information available from the complete genome, however, will facilitate much higher resolution association analyses. The BIGSDB platform has been designed to store such population-scale bacterial genome datasets with no constraint on the number of samples or loci that can be analysed.

### Reference gene-based analysis of genome data

As the number of new genomes from individual species or genera increases, a problem emerges as to how they can be compared. Existing tools [50-52] do not scale well to the challenge of handling multiple genomes at the scale that will be required in the near future. When more than a handful of comparisons are to be made, one method is to use single nucleotide polymorphisms (SNPs) to generate phylogenetic trees, identifying informative SNP markers to differentiate particular nodes [4,53-56]. Ongoing SNP discovery resulting in continual tree generation makes this approach cumbersome for large ongoing studies, and the effects of recombination on population structure may make any resulting tree misleading. A recent study used short-read Solexa data to investigate hospital transmission and intercontinental spread of MRSA ST-239 [5]. This study identified thousands of high-quality SNPs, allowing a phylogeny to be determined, but this approach depends on mapping the sequences to a high-quality reference genome of a related isolate. Such a method will not be generically applicable to large scale population studies with diverse genotypes.

Adopting a reference gene-based, as opposed to a reference genome-based, approach to analysis of whole or partial genomes avoids many of the problems associated with determining how related strains are to each other by existing genomic methods. As genes are generally the unit of selection, treating them as discrete units of analysis is valid and analogous to MLST where each unique allele at a locus is defined with an allele number. Instead of a seven locus typing scheme the technique can be applied to each of the coding sequences in the genome. Identifying gene-length regions of DNA by comparison to a database of reference sequences is conceptually straightforward, computationally simple, and highly accurate. The method scales well, without the need for reanalysis every time new variants are discovered or new isolates added to a dataset. This is a particular advantage for population datasets that can include many thousands of isolates.

The method can utilize the array of analysis tools developed or applicable for analysing MLST data [24,57-60] with the loci chosen to provide the level of discrimination required or to focus on particular aspects of the biology of the organism. A further advantage is that the method is additive to and fully compatible with existing sequence typing schemes, so that new isolates with genome data can easily be categorized and compared to the large isolate datasets that have been accumulated over many years and geographical locations.

### Intra-genus analysis of species groups

With the exception of those defining rRNA variation, most existing sequence typing and characterisation databases host data for one or a few related microbiological species. BIGSDB allows loci to belong to groups which could be related to their level of diversity or by their presence in specific organisms so it can be specified that, for example, a *N. meningitidis *isolate should be scanned against *Neisseria*-specific loci and against more widely applicable loci such as genes for DNA and RNA metabolism or protein folding which are found widely throughout the bacterial domain [61]. Such groupings allow a single database to hold information for a disparate range of species, with characterisation ranging from the genus to a finetype, as appropriate, depending on their similarity and the research question being addressed. The trans-genus *Streptococcus *database demonstrates how multiple MLST schemes can be accommodated within a single database, allowing such schemes to be cross-referenced and sequences exported for phylogenetic analysis. From the analysis of 43 Streptococcal genomes, 77 loci were found in all sequences using a nucleotide BLAST search with the *S. equi *genome [40] as a reference. The identification of these genes, without prior knowledge, allowed a ClonalFrame tree to be constructed showing the individual species as distinct clusters, very similar to the tree constructed from MLSA locus data. Many more loci would be expected to be found throughout the genus if the BLAST search for each locus used an expanding database of all known alleles rather than the single reference genome sequence, as used for the MLSA scheme incorporated in the same database. Alternatively, loci can be defined based on the translated protein sequences, enabling protein BLAST searches to be used. The relative insensitivity of nucleotide BLAST searches compared to those for proteins [41], however, is unlikely to be an issue for isolates belonging to the same or closely related species.

## Conclusions

Recent advances in sequencing technology have removed the collection of these data as a limiting step in the study of bacterial populations. It is now possible to undertake whole genome studies on multiple isolates and such limits of cost and speed as remain are likely to be breached in the very near future. The study of bacterial populations now faces the challenge of exploiting this rich source of inference, potentially from whole genomes of thousands of isolates, and to do this it will be necessary to collect structured representative samples of populations and to link precise provenance and phenotype information with the sequence data. The success of the MLST approach to bacterial isolate characterisation was greatly facilitated by the accessibility of the data via the Internet, which enabled community participation in the collection and analysis of the data. MLST also provided a hierarchical and structured approach to population analysis, as well as linking the sequence type to relevant phenotype and provenance information. BIGSDB replicates and extends this paradigm by enabling whole genomes, or fragments of them, to be archived and the data to be organised and interpreted by any number of schemes, which can comprise any number of loci. Using BIGSDB, genomic data can be used to characterise isolates in many different ways but it can also be efficiently exploited for evolutionary or functional studies. Permitting indexing of loci on a functional basis, by treating loci or groups of loci as independent units of analysis, opens the way for genome annotation to become a community-based process [62,63]. BIGSDB represents a freely available resource that will assist the broader community in the elucidation of the structure and function of the bacteria by means of a population genomics approach.

## Availability and requirements

**Project name**: BIGSDB

**Project home page**: http://pubmlst.org/software/database/bigsdb/

**Operating systems**: Linux/UNIX

**Programming language**: Perl, Javascript

**Other requirements**: Apache, PostgreSQL

**License**: GNU GPL

**Any restrictions to use by non-academics**: none

## Abbreviations

LIMS: Laboratory Information Management System; MLST: Multi-Locus Sequence Typing; MLVA: Multi-Locus VNTR Analysis; MRSA: Methicillin-Resistant *Staphylococcus auerus*; SNP: Single Nucleotide Polymorphism; ST: Sequence Type; VNTR: Variable Number Tandem Repeats

## Authors' contributions

MM and KJ conceived the design concept and wrote the manuscript. KJ developed the software. Both authors read and approved the manuscript.
